# Environmental hierarchy as the third dimension of nanomaterial transformation science

**DOI:** 10.1016/j.eehl.2025.100195

**Published:** 2025-10-28

**Authors:** Swaroop Chakraborty

**Affiliations:** School of Geography, Earth & Environmental Sciences, University of Birmingham, Birmingham, B15 2TT, UK

**Predicting nanomaterial (NM) fate requires more than structural snapshots and degradation curves. Current 2D models, which focus on space (structural change) and time (kinetics), provide valuable insights but remain incomplete. They cannot fully explain why identical NM behave differently across environments. Here, this *Comment* proposes a 3D framework that adds a critical third axis—environmental hierarchy—to capture transformations that are orthogonal to space and time.**

NM fate depends on its intrinsic properties and time, but is critically governed by the environment encountered. To date, most studies of NM transformations have emphasized two axes of variability, i.e., spatial and temporal [1,2]. While this 2D view is powerful, it leaves important blind spots, particularly in explaining why the same NM can appear inert in one environmental tier but highly reactive or toxic in another. In this *Comment*, environmental hierarchy is proposed as a fundamental dimension of NM transformation science. Here, “environmental hierarchy” denotes an ordered axis of tiers(or compartments)—atmosphere, water/soil, and biota—used to organise mechanisms. It does not imply a single obligatory sequence; NMs may enter any tier first, although sequential transitions are common and considered explicitly in this commentary.

## 1 The 2D paradigm and its limitations

Current models and experiments describe how NM change (e.g., aggregation, dissolution, surface oxidation) and when they change (timescales of those reactions), but often treat the environment merely as a backdrop. In practice, however, puzzling divergences are observed: the same NM may be inert in one medium yet reactive or toxic in another. For example, TiO_2_ nanoparticles generate reactive radicals under UV-rich air conditions, whereas in dark aquatic environments they sit largely unreactive [3]. These observations highlight that environmental context can dominate transformation pathways.

Upon release, engineered NM interact dynamically with their surroundings. Their transformation processes, including aggregation, dissolution, oxidation, sulfidation, or photo/enzyme reactions, are interdependent with environmental conditions and directly impact their mobility and bioavailability [4]. Crucially, these processes depend as much on environmental conditions like pH as on the particles themselves. However, most NM fate models implicitly assume that a particle's transformation is governed by its own properties (e.g., size) and time, with the medium only modulating rates [4,5]. In reality, changes in “where” (the media/biology) can introduce entirely new processes or eliminate others, creating transformation regimes that are mechanistically orthogonal to size or time. A key example is the adsorption of natural organic matter, which forms an eco-corona [6] that alters a particle's reactivity. Consequently, transformation pathways are dictated by a hierarchy of environmental interfaces. For instance, AgNPs dissolve rapidly in seawater via Cl^−^-mediated precipitation, a mechanism absent in freshwater [7]. Such tier-specific mechanisms cannot be reduced to spatial or temporal variables; they require explicit integration of the environmental hierarchy axis.

## 2 The third dimension: Environmental and biological hierarchy

**Fig. 1:**
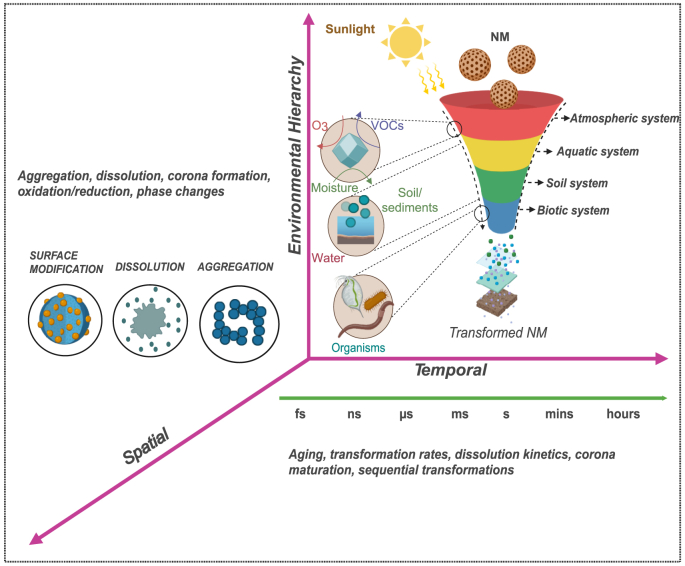
A 3D framework for NM transformations. NM transformations occur across three orthogonal axes: Spatial (e.g., aggregation, dissolution), Temporal (e.g., aging, transformation rates), and Environmental Hierarchy (sequential progression through atmosphere, water/soil, and biota). Each environmental tier imposes distinct transformation regimes: photochemical in air, eco-corona and ionic interactions in water/soil, and enzymatic or cellular transformations in organisms. The funnel illustration depicts this tiered cascade, highlighting transitions from physical to biological transformations, driven by environmental inputs such as sunlight, moisture, and biota. This 3D framework offers a predictive lens for understanding NM fate and guiding safer design.

It is proposed that NMs transformations should be conceived in three dimensions: spatial (structure/composition), temporal (kinetics), and a third axis—environmental hierarchy that spans physical media and biological complexity (e.g., atmosphere → water/soil → organism/biota). Each tier imposes distinctive chemical and biological rules (Fig. 1).

### 2.1 Atmosphere (air, reactive gases, VOCs)

Photochemistry, oxidative aging, and aerosol processes dominate. UV light and ozone can photo-oxidize NM surfaces or generate reactive radicals [3,8]. In fine aerosol droplets or dust, particles encounter high oxygen and radical fluxes, leading to reactions (e.g., O_3_ oxidation) absent in bulk water. Conversely, air offers low ionic strength and organic content, so things like electrostatic stabilization and organic coronas (common in water/soil) are minimal. In short, airborne particles face photolysis and gas-phase chemistries that are essentially orthogonal to the aqueous transformations typically studied.

### 2.2 Water and soil (aquatic/sediment)

In these media, ionic strength, dissolved organic matter, and mineral surfaces set the stage. Saline water accelerates or alters metal dissolution; for instance, chloride ions in seawater precipitate Ag^+^ as AgCl rather than allowing free Ag^+^ to accumulate [9]. Natural organic matter and extracellular polymers adsorb onto NM to form a so-called *eco-corona*, fundamentally changing reactivity and fate [10]. Aggregation and sedimentation become important: high ionic strength in saline water or soil pore fluids screens electrostatic repulsion and leads to clumping, while attachment to clays or organic sediments immobilizes particles. In other words, in water/soil, the particle's surface chemistry and colloidal stability—and thus transformations like dissolution or aggregation—are dominated by electrolytes and natural macromolecules.

### 2.3 Organisms/biota (biological interiors)

In this framework, “biota” is an umbrella tier spanning microbes, plants, and animals, encompassing extracellular fluids, membranes, and intracellular organelles. Transformation rules vary across these sub-tiers (e.g., bacterial extracellular enzymes vs. mammalian lysosomal processes); we therefore specify examples at the mechanism level. Blood, tissue fluids, and intracellular spaces are rich in proteins, enzymes, and cells. Particles become coated by a bio-corona (layer of adsorbed proteins and lipids) that is distinct from an eco-corona. Critically, living cells can actively transform NMs: lysosomal enzymes, oxidative bursts, and reductases modify particles in ways impossible abiotically. For example, particles gain bio-coronas and undergo enzymatic, oxidative, or pH-driven changes absent in abiotic media (e.g., peroxidase-driven degradation of graphene oxide) [11]. Critically, moving NMs from one tier to another resets their transformation rules. These transitions create phase-specific realities—the dominant thermodynamic drivers, kinetic bottlenecks, and even reaction products can change. Thus, the fate predicted by spatial–temporal models in one tier may be entirely invalid in another.

## 3 Why this is a true “dimension”

This conceptual shift is not merely a matter of semantics; it reflects a fundamental mechanistic independence that warrants treating environmental hierarchy as a true and orthogonal dimension of NM transformation science. While space/time describe how and when a NM changes, environmental hierarchy dictates if and by which rules it changes. For one, the environmental tier imposes constraints and opportunities that act independently of a particle's size or lifetime. A 50 ​nm particle will agglomerate quickly in seawater but stay suspended in clean air—the governing physics differs. Knowledge of the environmental compartment often has more predictive power than, e.g., minor size differences. For example, soil organic content largely determines carbon nanotube transport in soils [12], whereas those effects are irrelevant for airborne nanotubes. In essence, environmental hierarchy is orthogonal to spatial and temporal axes: it selects which subset of processes (chemical, physical, biological) are possible.

This third axis also brings immense predictive power. If we know the environmental tier (such as, agricultural soil vs atmospheric dust vs a cancerous tumour/or ecological organism), we can tailor our models to the dominant chemistry there. For example, the ion-mediated dissolution of Ag-NPs can be modelled accurately only by including chloride and sulphide chemistry in marine or sediment environments [13]. In organisms, one would instead include enzymatic kinetics and pH-dependent transformations. Importantly, many mechanistic pathways in one tier simply do not exist in another. The enzymatic oxidative degradation of graphene oxide by immune cells shares no mechanism with its ozone-induced oxidation in air. In practice, this means that a single NM may require different models for different media—unlike the space-time dimensions, which often reuse the same equations (e.g., kinetic decay curves). Materials such as pristine multi-walled carbon nanotubes, inert across multiple tiers, illustrate how the absence of transformation pathways drives persistence. Such cases emphasise the importance of the third axis in explaining not only when NM changes, but why some resist alteration and accumulate [14].

Finally, the hierarchy dimension is literally “one more thing” in modelling and experimental design. We can think of fate as a function *F (*space, time, environment hierarchy), rather than just *F* (space, time). Embracing this formality encourages new tools (tier-resolved fate maps, tier-specific degradation rate constants) and reminds us not to treat environmental context as a footnote.

## 4 Conclusion and implications for a dimension-aware future

Recognising environmental hierarchy as a genuine third axis—alongside structure and time—changes the way we must test, model, and design NMs. Risk assessment can no longer rely on single-medium assays; instead, it requires a tiered testing matrix that follows the real journeys of materials through atmosphere, water/soil, and biota. Such sequential exposures reveal hidden hazards—for instance, NM that appear inert in inhalation studies but dissolve or generate reactive species after contact with lung surfactant or aquatic runoff. Without stepping through these tiers, delayed or emergent risks remain invisible.

To make this vision operational, both experimental and computational tools must evolve. Laboratory protocols should verify particle state at each transition and feed those measurements directly into the next tier (discussed more as an operational consideration in SI). Modelling frameworks should be tier-resolved, “switching on” the chemistry that dominates in each setting—photochemistry in air, ionic strength and ligand binding in water and soils, enzymatic and pH-driven processes in organisms. This creates transparent chains of transformation rather than static snapshots. A detailed mapping of current OECD/EPA guidelines, 3D mappings, and practical notes are in Table S1.

For NM design, the implications are profound. Stability and degradability are not absolute properties but tier-specific specifications (SI). Nanomedicine already harnesses this logic with pH-responsive carriers that release drugs only in the acidic tumour microenvironment. Extending such intentional design to environmental technologies enables materials that are robust in use yet degrade predictably once released. There is a particular need to develop tier-resolved transformation profiles for emerging materials such as metal–organic frameworks (MOFs): stable when dry, but transforming in aqueous and biological tiers [15]. Framing NM fate as F(structure, time, environment hierarchy) provides clarity where confusion has long reigned. Dimension-aware nanoscience does more than sharpen prediction; it embeds sustainability at the design stage and ensures safety assessments keep pace with real-world complexity. The path forward is clear: test, model, and design along the axes that materials actually travel.

## CRediT authorship contribution statement

**Swaroop Chakraborty:** Writing – review & editing, Writing – original draft, Visualization, Validation, Supervision, Software, Resources, Project administration, Methodology, Investigation, Funding acquisition, Formal analysis, Data curation, Conceptualization.

## Declaration of competing interest

There is no conflict of interest to declare.
