# Supplementary material for: Environmental hierarchy as the third dimension of nanomaterial transformation science
Source: Eco Environ Health. 2025 Oct 28;4(4):100195. doi: 10.1016/j.eehl.2025.100195 (PMC12639430; doi:10.1016/j.eehl.2025.100195)
Supplement: Multimedia component 1 [file mmc1.docx]

**Supplementary Information**

**Environmental hierarchy as the third dimension of nanomaterial transformation science**

Swaroop Chakraborty^1*^

^1^School of Geography, Earth & Environmental Sciences, University of Birmingham, Edgbaston, Birmingham, B15 2TT, UK.

*[s.chakraborty@bham.ac.uk](mailto:s.chakraborty@bham.ac.uk)

**Table S1** Comparison of current OECD/EPA test guidelines with their limitations for nanomaterials and proposed tiered modifications.

| **Endpoint / route** | **Current guideline (OECD/EPA)** | **What it currently captures (2D focus)** | **Key gaps for nanomaterials & tiers** | **Concrete tiered modification we propose** | **Sources link** |
| --- | --- | --- | --- | --- | --- |
| **Dispersion & stability in media** | **OECD TG 318** (Dispersion stability of nanomaterials in simulated environmental media) | Colloidal stability under set aqueous conditions; agglomeration/settling tendencies | Focused on single-compartment water media; often omits prior **air** aging or subsequent **biota** exposure; limited coupling to dissolution/eco-corona | Add **sequential conditioning**: (i) air aging (UV/ozone) → (ii) freshwater/marine (ionic strength, DOM) with **eco-corona** formation → (iii) protein-rich media; track size, number, mass, and ion release at each tier | ([OECD](https://www.oecd.org/content/dam/oecd/en/publications/reports/2017/10/test-no-318-dispersion-stability-of-nanomaterials-in-simulated-environmental-media_g1g837a1/9789264284142-en.pdf?utm_source=chatgpt.com)) |
| **Dissolution / transformation** | **OECD GD (2020)** Testing dissolution & dispersion stability of nanomaterials | Dissolution rate and stability in aqueous media | Often decoupled from **eco-corona** and from ion-complex chemistry in saline/organic-rich matrices; no link to **organism** tier | Pair dissolution with **corona-state** (eco vs bio) and relevant ligands (Cl⁻, S²⁻); report **passaged rates** after each tier transfer (air→water/soil→biota) | ([OECD](https://www.oecd.org/content/dam/oecd/en/publications/reports/2020/07/guidance-document-for-the-testing-of-dissolution-and-dispersion-stability-of-nanomaterials-and-the-use-of-the-data-for-further-environmental-testing-and-assessment_988b598b/f0539ec5-en.pdf?utm_source=chatgpt.com)) |
| **Inhalation toxicity (subacute)** | **OECD TG 412** (28-day inhalation; revised to accommodate NMs) | Repeated aerosol exposure; respiratory endpoints | Limited **post-deposition** tiering (e.g., mucosal fluids → aquatic runoff); aerosol aging (UV/ozone) usually not required | Pre-age aerosol (UV/ozone) and require **post-exposure transfer** to simulated lung fluid and then to aquatic media to assess rain-out / secondary exposures | ([OECD](https://www.oecd.org/content/dam/oecd/en/publications/reports/2018/06/test-no-412-subacute-inhalation-toxicity-28-day-study_g1gh2939/9789264070783-en.pdf?utm_source=chatgpt.com)) |
| **Inhalation toxicity (subchronic)** | **OECD TG 413** (90-day inhalation; revised for NMs) | Longer-term respiratory toxicity | Same as TG 412 plus lack of **bio-corona** characterisation of deposited particles | Mandate characterisation of deposited particle **bio-corona** and subsequent **lysosomal** transformations; link to aquatic runoff tier if relevant | ([OECD](https://www.oecd.org/content/dam/oecd/en/publications/reports/2018/06/test-no-413-subchronic-inhalation-toxicity-90-day-study_g1gh293b/9789264070806-en.pdf?utm_source=chatgpt.com)) |
| **Algal growth inhibition** | **OECD TG 201** | Population growth effects over 72 h | Poor control of NM behaviour (agglomeration, shading, dissolution); media often not optimised for NM stability; no **eco-corona** accounting | Require NM **behavioural pre-tests** in test media; report number- and mass-based dose, PDI, dissolution; include **eco-corona pre-conditioning** and light regime relevant to **air→water** transitions | ([OECD](https://www.oecd.org/content/dam/oecd/en/publications/reports/2011/07/test-no-201-freshwater-alga-and-cyanobacteria-growth-inhibition-test_g1gh28f1/9789264069923-en.pdf?utm_source=chatgpt.com)) |
| **Daphnia acute immobilisation** | **OECD TG 202** | 24/48 h acute effects | Static tests can misrepresent exposure due to settling; dissolved vs particulate dose rarely resolved; no **tier carry-over** | Use **flow-through** or gentle mixing to maintain exposure; measure **separated fractions** (dissolved ions vs particles); include prior **eco-corona** step; report delivered dose (number/mass), size & ion release | ([OECD](https://www.oecd.org/content/dam/oecd/en/publications/reports/2004/11/test-no-202-daphnia-sp-acute-immobilisation-test_g1gh28f3/9789264069947-en.pdf?utm_source=chatgpt.com" \o "Test No. 202: Daphnia sp. Acute Immobilisation Test (EN))) |
| **Fish acute toxicity** | **OECD TG 203** | 96 h acute lethality | NM behaviour in tanks (agglomeration/sedimentation) often uncontrolled; no **prior tier** conditioning; no continuous characterisation | Require **time-resolved** characterisation (size/number/dissolution) during test; include **eco-corona** pre-conditioning (freshwater vs marine) and document light/UV exposure history | ([OECD](https://www.oecd.org/en/publications/2019/06/test-no-203-fish-acute-toxicity-test_g1gh28f5.html?utm_source=chatgpt.com)) |
| **Daphnia reproduction (chronic)** | **EPA OPPTS 850.1300** (Daphnid chronic) | Life-cycle endpoints over ≥21 d | Typically mass-only nominal dosing; limited tracking of **transformations** and **eco-corona** dynamics | Require **tier-aware dosing** (particle + ion), periodic re-characterisation, and **corona profiling**; include renewal strategy that preserves NM behaviour | ([US EPA](https://www.epa.gov/sites/default/files/2015-07/documents/850-1300.pdf?utm_source=chatgpt.com)) |
| **Cross-cutting: aquatic/sediment nano specific guidance** | **OECD Series on Testing & Assessment No. 317** (Aquatic & sediment toxicological testing of nanomaterials; 2nd ed., 2025) | NMs considerations across aquatic/sediment tests | Provides nanospecific advice but still largely **single tier**; limited operationalisation of **sequential** tier transfers | Embed **sequential tier modules** (pre-aging → eco-corona → organism exposure) and require reporting of **tier-resolved** endpoints | ([OECD](https://www.oecd.org/content/dam/oecd/en/publications/reports/2019/06/test-no-203-fish-acute-toxicity-test_g1gh28f5/9789264069961-en.pdf?utm_source=chatgpt.com)) |
| **EPA ecological effects (overview)** | **EPA Series 850** (Ecological Effects Test Guidelines) | Broad suite for aquatic/terrestrial taxa | Not tailored to NM tier transitions; limited requirements for behaviour/transformations characterisation | Add **nanomaterial annexes** specifying tiered pre-conditioning, dose metrics (number/mass/ion), and continuous characterisation across tiers | ([US EPA](https://www.epa.gov/test-guidelines-pesticides-and-toxic-substances/series-850-ecological-effects-test-guidelines?utm_source=chatgpt.com)) |

**S1. Operational triggers across tiers.** In practice, regime shifts are initiated when environmental variables cross colloidal or chemical thresholds. (i) **Ionic strength & valence:** aggregation accelerates once ionic strength exceeds a system-specific **critical coagulation concentration (CCC)**, which drops sharply in the presence of divalent cations (Schulze–Hardy behaviour); freshwater→seawater transitions often exceed these thresholds, producing rapid, sometimes irreversible aggregation^1,2^. (ii) **Ligands & complexing anions:** chloride and sulphide re-speciate silver, switching dominant pathways from oxidative dissolution to AgCl_x_​ complexation or Ag_2_​S formation, with large effects on bioavailability and toxicity^3,4^.(iii) **Organic macromolecules (eco-/bio-corona):** rapid adsorption of natural organic matter and biomolecules produces an interfacial corona **within minutes**, altering surface energy, dissolution and adhesion^5,6^. (iv) **pH & redox / enzymes:** acidic, enzyme-rich intracellular compartments favour dissolution or oxidative degradation (e.g., peroxidase-driven attack on graphene materials), whereas anoxic sediments favour reduction/sulfidation^7^.

## **S2. Implications for Testing, Safety and Design.** Viewing environmental hierarchy as a genuine dimension reshapes how we assess and design nanomaterials. In **risk assessment**, it suggests that regulatory testing must be tiered. Instead of testing a material in one “generic” media, assays should mimic real-world transitions: airborne exposure tests (e.g. inhalation models) should be followed by aquatic and then biological assays that capture rain-out and uptake. A “3D” testing matrix (atmosphere → water/soil → biota) will better reveal hazards. For example, an inhaled nanoparticle may be benign until it enters the lung surfactant (wet, protein-rich), at which point it dissolves or generates reactive species. Only by stepping through these tiers can we predict such delayed effects. To make this proposal more concrete, I provide in **Table S1** (Supplementary Information) a comparison of current OECD and EPA assays with the specific gaps they present for NM testing, and suggested modifications that embed the environmental-hierarchy axis. The analysis shows, for example, that while OECD TG 318 captures dispersion stability in water, it does not account for prior air aging or subsequent bio-corona formation. Similarly, inhalation guidelines (OECD TG 412/413) capture aerosol exposures but rarely consider transformations following deposition into lung fluids or aquatic runoff. **Table S1** highlights how a tier-aware approach -incorporating sequential pre-aging, eco- and bio-corona conditioning, and fractionated dose metrics (particle number, surface area, dissolved ions) -can enhance regulatory relevance. By explicitly mapping current versus proposed protocols, this supplementary table demonstrates how tiered testing could be operationalised within existing OECD/EPA frameworks. **Practical considerations.** In the lab, simulating sequential transitions (atmosphere → water/soil → biota) should use the least disruptive handling available (e.g., gentle deposition or low-shear transfers) and simple checks at each step to confirm the particle state (hydrodynamic size/number and dissolved fraction). For modelling, a pragmatic approach is to run **tier-specific** models and pass measured outputs from one tier as inputs to the next, recording assumptions and reporting ranges rather than single values. **Table S1** notes where these steps can be added to current OECD/EPA assays.

While adopting a tiered testing matrix (atmosphere → water/soil → biota) inevitably presents logistical and economic challenges - including the costs of running multiple assays, the requirement for specialised infrastructure and expertise facilities (e.g., inhalation chambers, aquatic mesocosms, and advanced cell culture models, advanced NM characterisation facilities such as advanced light source (synchrotron)^8^), and the difficulty of standardising protocols across laboratories and jurisdictions—acknowledging and addressing these hurdles is a necessary evolution for accurate and realistic risk assessment.

In **nanomedicine and precision therapy**, the hierarchy concept is already being exploited – sometimes unwittingly – by designing *environment-responsive* carriers. Tumours, for instance, have a mildly acidic extracellular pH and highly acidic lysosomes. Nanocarriers use pH-sensitive bonds or coatings that remain stable in blood (pH ~7.4) but cleave in the tumour microenvironment or intracellular compartments^9^. This is precisely using the third axis: the “organism” tier (tumour tissue) triggers transformations (e.g. drug release) that would not occur in normal tissue or *in vitro*. Thinking in three dimensions can extend this idea: we can intentionally design particles to be inert in the manufacture and transport phase but activated/degraded in specific environmental compartments (e.g. sunlight-activated antimicrobials in air or enzyme-cleavable coatings in soil bacteria). For chemists designing nanomaterials, this demands a paradigm shift: stability must be defined per environmental compartment, not universally. Regulatory agencies (e.g., OECD, EPA) should mandate tiered testing (atmosphere → water → organisms/biota) to replace single-medium assays.

For **sustainable nanotechnology**, dimension-aware design is paramount. Ideally, a NM might be stable under its use conditions (perhaps in an inert gas or manufacturing solvent) but environmentally degradable once released^10^. For instance, one could engineer a particle that resists oxidation and aggregation in dry manufacturing (air) but dissolves readily in the alkaline, oxidizing conditions of certain waste waters. Conversely, we may avoid materials that are predicted to be persistent in every tier (e.g. fully hydrophobic nanoparticles that never dissolve or biodegrade in air, water, or organism). There is a particular need to develop **tier-resolved transformation profiles** for emerging materials such as **metal–organic frameworks (MOFs)**, which can appear stable under dry, inert conditions yet undergo hydrolysis, linker exchange, or secondary-phase formation in aqueous and biological tiers. This explicitly tiered view aligns with **Safe-and-Sustainable-by-Design** principles for MOFs^11–13^. In effect, sustainable design means specifying the desired transformation pathway in each environmental compartment – a fully 3D specification.

**References:**

1 L. Wang, X. Yang, Q. Wang, Y. Zeng, L. Ding and W. Jiang, *Journal of Environmental Sciences*, 2017, **51**, 248–255.

2 N. T. Loux, Y. S. Su and S. M. Hassan, *Int J Environ Res Public Health*, 2011, **8**, 3562.

3 C. Levard, S. Mitra, T. Yang, A. D. Jew, A. R. Badireddy, G. V. Lowry and G. E. Brown, *Environ Sci Technol*, 2013, **47**, 5738–5745.

4 C. Levard, E. M. Hotze, G. V. Lowry and G. E. Brown, *Environ Sci Technol*, 2012, **46**, 6900–6914.

5 F. Abdolahpur Monikh, L. Chupani, I. Karkossa, Z. Gardian, D. Arenas-Lago, M. von Bergen, K. Schubert, V. Piackova, E. Zuskova, W. Jiskoot, M. G. Vijver and W. J. G. M. Peijnenburg, *NanoImpact*, 2021, **22**, 100315.

6 Y. Xu, X. Wang, J. P. van der Hoek, G. Liu and K. M. Lompe, *Environ Sci Technol*, 2025, **59**, 1822.

7 R. Kurapati, C. Martìn, V. Palermo, Y. Nishina and A. Bianco, *Faraday Discuss*, 2021, **227**, 189–203.

8 S. Chakraborty, S. Britto, M. Gomez-Gonzalez, A. G. Buzanich and I. Mikulska, *Cell Rep Phys Sci*, 2025, **0**, 102806.

9 R. G. Thomas, S. P. Surendran and Y. Y. Jeong, *Front Mol Biosci*, 2020, **7**, 610533.

10 S. Chakraborty, D. Menon, I. Mikulska, C. Pfrang, D. Fairen-Jimenez, S. K. Misra and I. Lynch, *Nature Reviews Materials 2025*, 2025, 1–3.

11 D. Menon and S. Chakraborty, *Frontiers in Toxicology*, 2023, **5**, 1233854.

12 S. Chakraborty, B. Ibrahim, P. Dhumal, N. Langford, L. Garbett and E. Valsami-Jones, *Journal of Hazardous Materials Letters*, 2024, **5**, 100127.

13 P. Dhumal, P. Bhadane, B. Ibrahim and S. Chakraborty, *Green Chemistry*, 2025, **27**, 3815–3850.
